# Engineering motile aqueous phase-separated droplets via liposome stabilisation

**DOI:** 10.1038/s41467-021-21832-x

**Published:** 2021-03-15

**Authors:** Shaobin Zhang, Claudia Contini, James W. Hindley, Guido Bolognesi, Yuval Elani, Oscar Ces

**Affiliations:** 1 Department of Chemistry, Molecular Sciences Research Hub, Imperial College London, 82 Wood Lane, London, W12 0BZ UK; 2 Department of Chemical Engineering, Exhibition Road, Imperial College London, London, SW7 2AZ UK; 3 fabriCELL, Molecular Sciences Research Hub, Imperial College London, 82 Wood Lane, London, W12 0BZ UK; 4 Institute of Chemical Biology, Molecular Sciences Research Hub, Imperial College London, 82 Wood Lane, London, W12 0BZ UK; 5 Department of Chemical Engineering, Loughborough University, Loughborough, LE11 3TU UK

**Keywords:** Cellular motility, Drug delivery, Phospholipids, Polymers

## Abstract

There are increasing efforts to engineer functional compartments that mimic cellular behaviours from the bottom-up. One behaviour that is receiving particular attention is motility, due to its biotechnological potential and ubiquity in living systems. Many existing platforms make use of the Marangoni effect to achieve motion in water/oil (w/o) droplet systems. However, most of these systems are unsuitable for biological applications due to biocompatibility issues caused by the presence of oil phases. Here we report a biocompatible all aqueous (w/w) PEG/dextran Pickering-like emulsion system consisting of liposome-stabilised cell-sized droplets, where the stability can be easily tuned by adjusting liposome composition and concentration. We demonstrate that the compartments are capable of negative chemotaxis: these droplets can respond to a PEG/dextran polymer gradient through directional motion down to the gradient. The biocompatibility, motility and partitioning abilities of this droplet system offers new directions to pursue research in motion-related biological processes.

## Abstract (web summary)

Bio-mimetic motion has been hard to achieve due to a lack of biocompatible conditions. Here, the authors report the creation of a liposome-stabilised aqueous PEG/dextran Pickering-like emulsion system with motion induced by the Marangoni effect and characterised by negative chemotaxis.

## Introduction

The development of motile cell-mimetic compartmentalised systems is receiving increasing interest due to their potential in applications including targeted drug delivery^1–3^ and environmental restoration^4,5^. The fact that controlled motility and chemotaxis is a behaviour that is considered to be one of the hallmarks of life has also made it a focus in the endeavour to construct artificial cells that mimic biological cells in form and function^6,7^. To propel an object, various strategies have been exploited such as bubble propulsion^8–10^, diffusiophoresis^11–15^, magnetic force^16,17^ and the Marangoni effect^18–20^. Among these methods, the Marangoni effect is a commonly used way to induce motion since it can be easily achieved^3^. The asymmetric distribution of interfacial tension (IFT) around a droplet spontaneously generates a Marangoni flow on the droplet surface, driving its motion. A typical example of the Marangoni effect can be achieved by adopting a water/oil system, in which a water or oil drop can move in an oil or aqueous phase respectively by responding to changes in the droplet IFT^3,21–23^. For example, Xiao et al.^3^ reported that by adding photo-active surfactant precursors to an oil droplet, motion can be produced in an aqueous phase if one side of the oil droplet is irradiated by a light source to initiate a photo-responsive chemical reaction and then generate an asymmetrical distribution of IFT. Li et al.^23^ reported that water/ethanol droplets can achieve motion in an oil phase containing squalene/monoolein. In their system, water/ethanol droplets transform into Janus droplets in the oil phase by separating into water-rich and ethanol-rich part components. Monoolein holds an affinity to the ethanol-rich part, which leads to IFT difference between the water-rich part and the ethanol-rich part, generating a Marangoni flow and subsequent motion. However, with these water/oil systems, biocompatibility is an inevitable problem for those due to the existence of oil phases, which limits their applications in biotechnological settings. In contrast, water/water systems are ideal candidates for bio-related research because of their intrinsic biocompatibility. However, there are few examples about using water/water systems to produce motile droplets, and the stability and controllability of those motile water/water systems are inadequate^24,25^. Thus, there is a need to develop novel stable and controllable motile systems based on water/water systems.

The aqueous two-phase system (ATPS) is a common water/water system that forms when two polymers (such as polyethylene glycol (PEG) and dextran (DEX)) are co-dissolved in water^26,27^. In the aqueous solution, the polymer molecules segregate at high concentrations due to the poor entropic gain from polymer mixing. This causes separation into two phases (affected by pH, temperature and molecular weight of polymers)^28–30^. Both aqueous phases in ATPS have similar properties with the cell cytoplasm such as high viscosity as they are all composed of macromolecule assemblies produced through phase separation^31^. Apart from that, these two phases usually hold different physical and chemical properties, the difference of which allows their exploitation for separation and purification of biomolecules^27^. For example, natural proteins, DNA and urease prefer to diffuse into the DEX-rich phase while denatured proteins normally accumulate in the PEG-rich phase^32,33^. It is also reported that compartmentalisation can be accomplished in a liposome-stabilised ATPS-based emulsion system^34^, which provides a new method for designing artificial cells and organelles. Therefore, ATPS is an attractive system for many bio-related areas such as molecule separation^35,36^ and control of biomolecular reactions^37^.

Here, we show a biocompatible motile emulsion droplets system by combining the Marangoni effect with PEG/DEX ATPS. To stabilise these emulsion droplets, PEGylated liposomes are used as a droplet stabiliser. By introducing a PEG/DEX polymer gradient into this emulsion system, the aqueous droplets achieve motion as a result of the Marangoni effect. This motion is modulated by the liposomal stabiliser: in response to the PEG/DEX polymer gradient, the liposome coating will gradually become asymmetric generating an IFT imbalance across the droplet that weakens the Marangoni effect. Such an effect acts as a dampener, increasing the controllability of droplet motion. Since both its lumen and outer environment are fully aqueous, and possess cytoplasm-like properties, this motile droplet not only can act as a carrier for cargo delivery within biological environments but also serve as a platform to research motility-related cellular phenomena.

## Results

### Droplet structure and stability

**Fig. 1:**
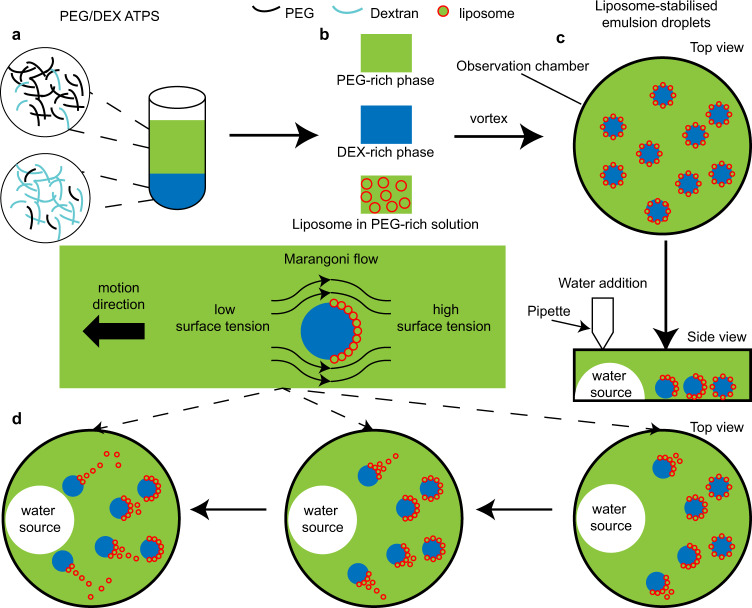
Engineering motile aqueous phase-separated droplets via liposome stabilisation. **a** PEG (polyethylene glycol)/DEX (dextran) solution separates into PEG-rich and DEX-rich phase to form ATPS (aqueous two-phase system). **b** Preparation of liposome in PEG-rich solution. **c** Preparation of emulsion droplets by mixing PEG-rich phase, DEX-rich phase and liposomes via vortex. **d** Droplets achieve directional motion towards the water source in response to a polymer gradient caused by the addition of water.

The ATPS in our research consists of PEG (10 wt/wt %), dextran (16 wt/wt %), and water (74 wt/wt %). After phase separation, two clear phases can be obtained: the upper is a PEG-rich phase while the lower is a DEX-rich phase^37^ (Fig. 1 and Supplementary Table 1). Water-in-water emulsions can be obtained by simply mixing PEG-rich phases and DEX-rich phases via vortex. In our case, droplets consisting of a DEX-rich (dispersed phase) are immersed in a PEG-rich phase (continuous phase). To verify the emulsion bulk stability, a UV-vis spectrometer was used to monitor the emulsion turbidity by measuring the absorbance of formed emulsions. We take the ratio of measured absorbance at each time point to the absorbance at 0 h as normalised absorbance to better analyse changes in particle size. The emulsion absorbance decreases quickly with time when an emulsion was made by simply mixing two aqueous phases (Supplementary Fig. 1), indicating the instability of the generated emulsion. Emulsion droplets are driven to merge to decrease the total surface energy. As a result, a turbid emulsion will gradually reseparate into two clear aqueous phases, causing a decrease in absorbance. Such experiments confirm the necessity of an emulsion stabiliser to generate stable droplets from PEG/DEX solutions.

**Fig. 2:**
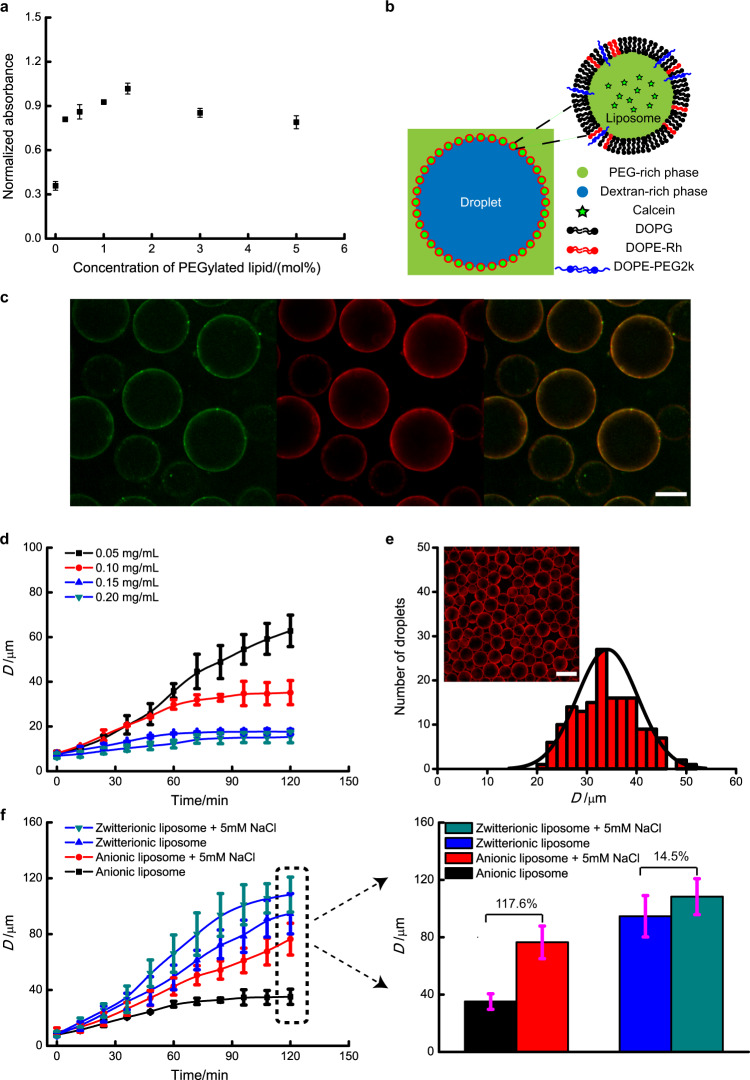
Characterisation of droplet morphology and stability. **a** Normalised absorbance (at 650 nm) 8 h after emulsion formation. If emulsions stay stable, it should remain turbid during observation, i.e. the normalised absorbance remains unchanged. A decrease in normalised absorbance can be observed for unstable emulsions. Error bars represent 1 s.d. (*n* = 3) for each data set. **b** Illustration of a liposome-stabilised droplet. DOPG, DOPE-Rh and DOPE-PEG2k corresponds to 1,2-dioleoyl-sn-glycero-3-phospho-(1′-rac-glycerol) (sodium salt), 1,2-dioleoyl-sn-glycero-3-phosphoethanolamine-N-(lissamine rhodamine B sulfonyl) (ammonium salt), and 1,2-dioleoyl-sn-glycero-3-phosphoethanolamine-N-[methoxy(polyethylene glycol)-2000] (ammonium salt), respectively. **c** Droplets stabilised by Calcein-containing (5 mM) liposomes. From left to right, Calcein channel, Rhodamine channel and an overlay of the calcein and rhodamine channels. Colours were added via Image J. Liposome solution is diluted after size exclusion, which leads to the large droplet size. Scale bar is 50 μm. **d** Effect of liposome concentration on droplet stability. D is droplet diameter. Error bars represent 1 s.d. (*n* = 50) for each data set. **e** Droplet size distribution for stable droplets (stabilised with DOPG liposomes). The inset picture corresponds to droplets at 120 min for 0.10 mg/mL in (**d**). Scale bar is 50 μm. **f** Effect of liposome type and salt addition on droplet stability. Droplet diameter at 120 min was extracted to draw a histogram in order to show the difference of salt addition on different liposome-stabilised droplets. Error bars represent 1 s.d. (*n* = 50) for each data set.

We chose liposomes for PEG/DEX droplet stabilisation as they have been shown to be a good stabiliser for ATPS^34^. The liposomes used in our research are primarily composed of charged DOPG phospholipids because the electrostatic repulsion between the DOPG liposomes is beneficial to producing stable droplets. We then investigated the effect of liposome composition on emulsion stability by varying the molar concentration (0–5 mol%) of PEGylated lipids in liposomes. The absorbance of ATPS emulsions stabilised by unPEGylated DOPG liposomes shows the same trend with time as that of the emulsion made without liposomes (Supplementary Fig. 1), indicating that unPEGylated liposomes are unsuitable for emulsion droplet stabilisation, leading to the coalescence of droplets. We attributed this phenomenon to the partition preference of unPEGylated liposomes to the DEX-rich phase (droplet lumen)^34^. To adjust liposome partition preference and to provide additional steric stabilisation of droplets, we added different amounts of DOPE-PEG2k lipids into the liposome formulation. With PEGylated liposomes, the emulsion absorbance shows a limited change in 8 h (Supplementary Fig. 1), indicating that the emulsions are stable during the observational period. To quantitively analyse emulsion stability, the absorbance at time *t* = 8 h was normalised with respect to the initial value at time *t* = 0 h. Figure 2(a) shows that emulsion stability increases with the concentration of PEGylated lipids and reaches a maximum at 1.5 mol%. After this point, a further increase in the PEGylated lipid concentration will lead to a gradual decrease in emulsion stability. With the increase of PEGylated lipid concentration, liposome partitioning preference gradually changes from the DEX-rich phase to PEG-rich phase^34^. When the concentration of PEGylated lipid is below 1.5 mol%, increased partition preference to the PEG-rich phase will cause liposomes to gradually move from the DEX-rich phase (droplet lumen) to the PEG-rich phase of the droplet interface, resulting in increased emulsion stability. Once the concentration is above 1.5 mol%, liposomes partition further into the PEG-rich phase, leading to a decreased emulsion stability. Hereafter, it can be assumed that 1.5 mol% PEGylated liposomes are used to produce all further emulsions unless explicitly stated.

It has been reported that PEGylated liposomes will remain at the droplet interface and form a barrier^34^ in liposome-stabilised emulsions (Fig. 2b). To verify the droplet structure, we added fluorescent DOPE-Rh lipids in liposomes to monitor their position. Calcein, a water soluble dye, was encapsulated in rhodamine-dye PEGylated liposomes to further help locate and monitor if the liposomes remain intact. These liposomes were purified via size exclusion chromatography before use to remove any unencapsulated calcein (Supplementary Fig. 2). The resulting purified liposome solution was then used to form emulsion droplets by mixing it with ATPS, and the emulsion observed using a microscope under calcein and rhodamine channels. As shown in Fig. 2c, the edge of the droplets is bright while the lumen is relatively dark, which means that liposomes remain at the droplet surface when stabilising the emulsion. In addition, the image obtained from the calcein channel is overlapped with the image obtained in the rhodamine channel, which means calcein fluorescence is localised on the droplet surface. The retention of calcein on the droplet surface indicates that the dye-loaded liposomes remain intact when forming a barrier. Hereafter, all liposomes contain DOPE-Rh to aid observation via fluorescence microscopy, and droplet pictures are captured in the rhodamine channel unless explicitly stated.

To investigate droplet stability, we prepared different emulsions by varying the liposome concentration from 0.05 mg/mL to 0.20 mg/mL, and then measured the droplet size every 12 min within a period of 120 min (Supplementary Fig. 3 and Supplementary Fig. 4). As shown in Fig. 2d, the initial droplet size is nearly the same despite the liposome concentration being changed. This is because the initial droplet size is only dependent on the mixing strength during the emulsion formation. Droplet size increases with time, and a lower liposome concentration corresponds to a faster increase in droplet size causing a significant difference in droplet size after 2 h. At this time, the droplet diameter for 0.05 mg/mL is nearly double that for 0.10 mg/mL and nearly four times bigger than that for 0.15 and 0.20 mg/mL. Moreover, for concentrations above 0.05 mg/mL, a plateau region can be observed, and the time needed for reaching the plateau stage decreases with liposome concentration (72 min for 0.15 or 0.20 mg/mL versus 96 min for 0.10 mg/mL). The assumption was made that a minimum coverage is necessary to create stable droplets. This coverage can be defined with the liposome surface concentration that is the ratio of the number of liposomes at droplet surface to droplet surface area (see section 5 in Supplementary Information). At the beginning of the observation, the surface concentration is not high enough to prevent droplet coalescence although a liposome barrier can be formed around DEX-rich droplets at the interface. As a result, frequent droplet coalescence occurs after emulsion formation with a consequent rapid increase of droplet size. The surface concentration increases as droplets merge, and droplets become stable enough to resist coalescence once the surface concentration is above the threshold. As a result, the droplet size remains unchanged (plateau stage in Fig. 2d). When the liposome concentration increases from 0.05 mg/mL to 0.20 mg/mL, the surface concentration at 0 min increases as well, thus fewer coalescence events are needed for reaching the threshold surface concentration and less time is needed to reach the plateau. Figure 2(e) shows the droplet size distribution when droplets become stable (at 120 min for 0.10 mg/mL). In addition, Supplementary Fig. 5 shows that droplet stability increases with liposome size when the same number of liposomes were used for stabilisation.

We then further investigated the effect of liposome composition on droplet stability. Liposomes containing zwitterionic (DOPC) or anionic (DOPG) lipids were used to generate emulsion droplets with the liposome concentration fixed at 0.10 mg/mL. Figure 2(f) demonstrates that the size of droplets stabilised by anionic liposomes (hereafter referred as anionic droplets) reaches a stable stage within 96 min after formation while that of droplets stabilised by zwitterionic liposomes (hereafter referred as zwitterionic droplets) keeps changing even 2 h after formation, showing that anionic droplets are more stable at the same liposome concentration. We attribute this stability variance to the difference in the charge between anionic lipids and zwitterionic lipids. When liposomes are composed of anionic lipids, electrostatic repulsion will exist between the liposomes, and this repulsion will cause an increased distance between liposomes at the droplet interface. Therefore, compared with zwitterionic droplets, less liposomes are needed to cover the surface of anionic droplets, resulting in less coalescence events (and less time) necessary to produce stable droplets. To further confirm the electrostatic stabilisation of droplets with charged liposomes, the emulsion stability was investigated in the presence of salt. Sodium chloride (NaCl) was added into the PEG-rich phase before mixing, with the preparation of emulsions as previously stated. When NaCl was added into the emulsion, the diameter for anionic droplets at 120 min significantly increases (117.6% increase, Fig. 2f) whilst the zwitterionic droplets’ diameter undergoes a much smaller increase (increased 14.5%, Fig. 2f). This indicates that NaCl has a much bigger effect on the stability of anionic droplets. As mentioned above, electrostatic repulsion between anionic liposomes is beneficial for stabilising droplets. However, salt addition shields the electrostatic repulsion between anionic liposomes due to charge screening effect within the emulsion. Thus, the stabilisation passes from electrostatic to steric in nature with a significant reduction of the distance between anionic liposomes. This greatly reduces the surface area that can be covered at a fixed liposome concentration and leads to a large reduction in the stability of anionic droplets. As a consequence, increased merging occurs within the same time duration, causing a large change in droplet diameter at 120 min. In comparison, the electrostatic interactions between zwitterionic liposomes are reduced. The small decrease in droplet stability can be accredited to the effect of water removal induced by salt addition rather than a change in the nature of droplet stabilisation. The addition of NaCl in PEG-rich removes water between liposomes^38^, and this reduces the distance between liposomes and subsequently, the droplet stability.

### Directional droplet motion

**Fig. 3:**
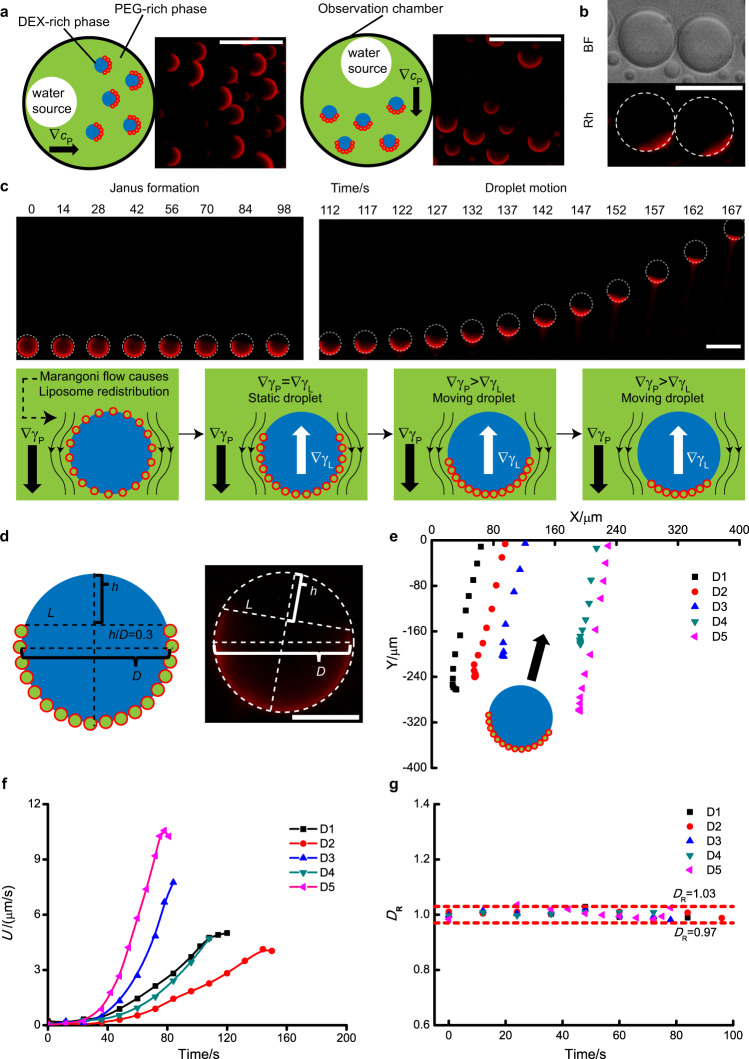
Formation of Janus droplets and subsequent droplet motion. **a** Janus conformation aligns with the position of water addition. Pictures were extracted from Supplementary Movies 1 and 2 and cropped for better presentation. $\nabla _{C_{\mathrm{P}}}$ is the polymer gradient. **b** Droplet structure under bright field (BF) or Rhodamine fluorescence channel (Rh). **c** Janus formation and droplet motion. In respond to water gradient, the droplet gradually transformed into a Janus droplet. The morphology change under the rhodamine channel is due to the redistribution/desorption of liposomes since Fig. 3b shows that droplets stay spherical under bright field despite the Janus morphology under the rhodamine channel. When the ratio of *h* to *D* reached ∼0.3, the droplet starts a directional motion towards the water source, with a characteristic tail of liposomes due to liposome desorption from the droplet surface during the motion. ∇_*γ*P_ and ∇_*γ*L_ are the IFT gradients caused by polymer gradient and liposomes, respectively. Frames were extracted from Supplementary Movie 1 and cropped for better presentation. **d** Illustration for the Janus droplet with *h*/*D* = 0.3. The concentric circle and the line L were drawn as auxiliary lines, and then a line h that is perpendicular to the line L was drawn. The diameter of the concentric circle and the length of line is *D* and *h,* respectively. **e** Trajectories of moving droplets. D1–D5 are representative droplets chosen from Supplementary Movie 1. Arrow corresponds to the direction of movement. **f** Corresponding velocity (U) and **g** diameter change (*D*_R_, the ratio of droplet diameter at different time points to its average diameter during motion) for droplets in (**d**). The time point when directional motion starts were chosen as time zero for each droplet. Scale bar is 50 μm for (**a**–**c**) and 20 μm for (**d**).

DOPG droplets were prepared with a 0.10 mg/mL liposome concentration and then transferred into an observation chamber. The emulsion was then left for 1 h to equilibrate. We then added 50 μL water to one side of the chamber (see Fig. 3a). This led to the creation of a polymer gradient ($\nabla _{C_{\mathrm{P}}}$). In response to this, we observed that the PEG/DEX emulsion droplets transformed from homogenously fluorescent droplets into asymmetric Janus droplets, where only the region of droplets further away from the water source is fluorescent (Fig. 3a, Supplementary Movie 1–3). We note that Janus droplets aligned with the relative location of the water source, and along the polymer gradient: the droplet hemisphere closer to the water source (front side) is dark while the other part (rear side) is bright, as shown in Fig. 3a. Figure 3b shows Janus droplets remain intact spherical structure under bright field, which means the transformation of PEG/DEX droplets is caused by changes in the liposome coating on the surface of the PEG/DEX emulsion droplets. The total fluorescence intensity of liposome coating during the transformation in Janus structures was roughly constant (Supplementary Fig. 6). This indicates that the formation of Janus droplets is due to the rearrangement of liposomes on the droplet surface rather than the loss of liposomes in PEG-rich phase. As shown in Fig. 3c, d, this transformation can be monitored by observing how the height of a non-fluorescent portion of the droplet (*h*) changes during the structural transformation. We then define *ε* as the ratio of *h* to *D* (droplet diameter) to quantitatively characterise the Janus structure for each droplet.

As a control, we found that adding a PEG-rich phase instead of a water solution simply causes limited droplet merging within the local vicinity, while adding more of the DEX-rich phase eliminates a great number of emulsion droplets within the chamber (Supplementary Fig. 7, Supplementary Movies 4 and 5). No formation of asymmetric Janus droplets or motility was observed in either case.

We found that motility started when the droplets reached a Janus structure corresponding to ∼*ε* ~ 0.3 (Fig. 3d). At this point, droplets started moving toward the water source with a fluorescent tail following at the rear side of droplets indicating a loss of droplet-associated liposomes (Fig. 3c). Figure 3e shows that the trajectories of moving droplets are directional, and droplet trajectories are nearly parallel to each other (see supplementary discussion in Supplementary Information for detail). Moreover, the direction of movement aligned with the axis of symmetry of the Janus structure. As shown in Fig. 3f, the droplet velocity increased with time during motion. In addition, the droplet diameter did not change significantly during motion, as shown in Fig. 3g.

The Marangoni effect can be used to explain the topology changes and the directional motion observed (lower panel in Fig. 3c). It has previously been reported that in the absence of liposomes the formation of a polymer gradient creates an IFT gradient ($\nabla \gamma _{\mathrm{P}}$) since the IFT between a PEG-rich phase and a DEX-rich phase changes with the polymer content in the ATPS^39^. As a result, the two poles of the droplet close to and away from the water gradient experience different IFTs. This imbalance induces a Marangoni flow on the droplet surface (from front side to rear side). In our experiments, where liposomes reside at the interface, this flow leads to the change in the liposome coating on the droplets surface and a change from a homogeneously coated droplet to one with an asymmetrical distribution. The asymmetrical liposome coating will cause an IFT gradient (∇*γ*_L_) in the opposite direction that balances the water-induced IFT gradient, leading to the immobilisation of the droplet interface. As a result, droplets cannot initially achieve motion. Once the *ε* of Janus structure reaches ∼0.3, the ∇*γ*_L_ cannot balance ∇*γ*_P_, so droplets begin their directional motion towards the water source. As the liposomes accumulate at the droplet rear end, the repulsive electrostatic/steric interactions between liposomes increase until the particles are expelled from the interface. When the droplet moves with respect to the surrounding medium, a trail of liposomes accompanies the droplet motion (Supplementary Movies 1–3). Such a mechanically forced desorption of particles from a liquid–liquid interface has been observed for nanoparticles at oil–water interface systems^40^.

### Analysis of d**r**oplet motion

For a qualitative analysis of droplet motion, we can model the droplet surface as a sharp (not diffuse) interface with an IFT *γ* that depends on the total polymer concentration *c*_P_ and the surface concentration *σ* (see section 8 in Supplementary Information). Cartesian (**e**_*x*_, **e**_*y*_, **e**_*z*_) and spherical (**e**_*r*_, **e**_*θ*_, **e**_*ϕ*_) coordinate systems are defined with the origin at the droplet centre and *z* axis parallel to the total polymer concentration gradient, so that $\nabla c_{\mathrm{P}} = \frac{{{\mathrm{d}}c_{\mathrm{P}}}}{{{\mathrm{d}}z}}{\boldsymbol{e}}_z$. Since the IFT *γ* increases with *c*_P_^39^, in the absence of liposomes droplets would migrate towards lower polymer concentration regions due to the Marangoni effect at a speed of

1 $${\mathbf{U}} = \frac{{ - D}}{{2\left( {2\mu _{\mathrm{o}} + 3\mu _{\mathrm{i}}} \right)}}\frac{{{\mathrm{d}}c_{\mathrm{P}}}}{{{\mathrm{d}}z}}{\mathrm{{\Gamma} }}{\mathbf{e}}_z$$

where *μ*_o_(*μ*_i_) is the viscosity of the outer (inner) phase and both $\frac{{{\mathrm{d}}c_{\mathrm{P}}}}{{{\mathrm{d}}z}}$ and ${\mathrm{{\Gamma} }} \simeq \left( {\frac{{\partial \,\gamma }}{{\partial \,c_{\mathrm{P}}}}} \right)$ are assumed to be constant during droplet motion on the time scale of experimental observation. Following the work of Levan and co-workers^41,42^, Eq. (1) can be derived by solving the Stokes equations for the dispersed and continuous phase and imposing the balance of tangential stresses at the droplet surface $\tau _{r\theta }^{({\mathrm{i}})} - \tau _{r\theta }^{\left( {\mathrm{o}} \right)} = \nabla \gamma \cdot {\mathbf{e}}_\theta$, where $\tau _{r\theta }^{({\mathrm{i}})}$ and $\tau _{r\theta }^{\left( {\mathrm{o}} \right)}$ are the tangential stress at the droplet surface exerted by the inner and outer phase, respectively (see section 8 in Supplementary Information). The same theoretical framework has been used to predict the behaviour of many other Marangoni-driven droplet systems^43–48^.

**Fig. 4:**
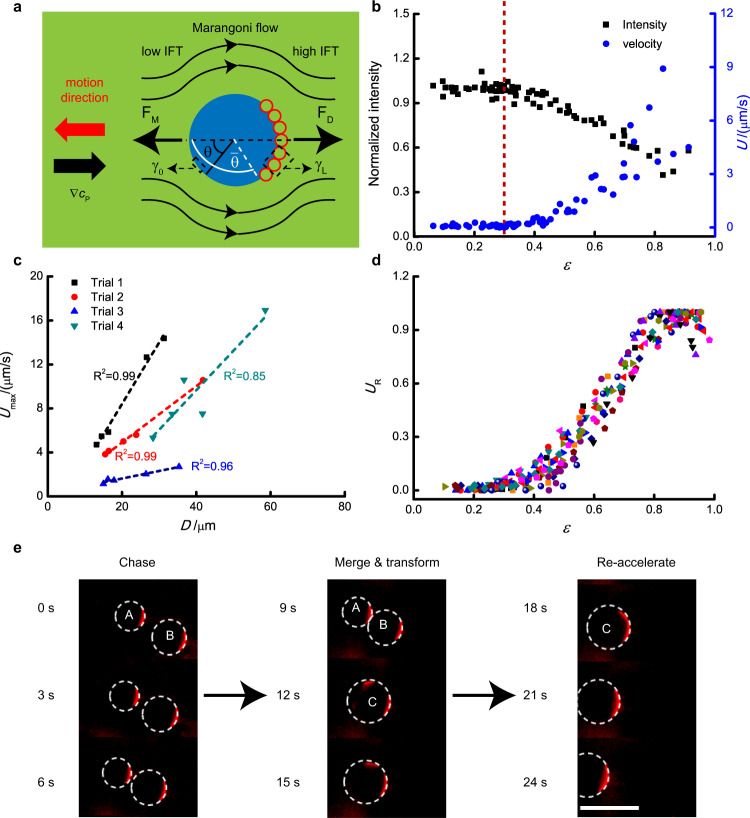
Mechanism behind motion. **a** Illustration for the Marangoni effect. *F*_M_ is the force induced by Marangoni stresses, while *F*_D_ is the viscous drag force. *γ*_L_ and *γ*_0_ is the interfacial tension between PEG-rich and DEX-rich phase with or without the presence of liposomes. **b** The total intensity of liposome coating and corresponding droplet velocity (*U*) for different droplets. **c** The relationship between maximum velocity (*U*_max_) and droplet diameter (*D*). The difference in the slope for different batches is due to the variations in polymer gradients. **d** The relationship between normalised velocity (*U*_R_, the ratio of droplet velocity at different time points to its maximum velocity) and the value of *ε* (*h*/*D*). 20 droplets from four trials were chosen for analysis. **e** Chasing and merge process for two droplets with different diameter. Pictures were extracted from Supplementary Movie 6 and cropped for better presentation. Scale bar is 50 μm.

In presence of liposomes, droplet motion still occurs in the same direction but liposomes accumulate at the rear end of the droplet due to fluid motion at the droplet interface, as shown in Fig. 3c. To a first approximation, this configuration can be modelled as shown in the schematic in Fig. 4a. In the region $\theta$ $< \,\bar \theta$, there are no absorbed liposomes and the corresponding IFT can be denoted as $\gamma _0\left( \theta \right) = \gamma (c_{\mathrm{P}}\left( \theta \right),\sigma = 0)$, whereas in the region $\theta \ge \bar \theta$, there are absorbed liposomes at a surface concentration *σ* and the corresponding IFT in this region is denoted as $\gamma _{\mathrm{L}}\left( {\theta ,\sigma } \right) = \gamma (c_{\mathrm{p}}\left( \theta \right),\sigma )$. Due to the spontaneous absorption of liposomes at the droplet interface, the IFT decreases with the liposome surface concentration and hence $\gamma _{\mathrm{L}}\left( {\bar \theta } \right) \,< \,\gamma _0(\bar \theta )$. As a result, the liposome distribution induces Marangoni-stresses at the droplet interface, proportional to ${\mathrm{{\Delta} }}\gamma (\sigma ) = \gamma _0\left( {\bar \theta } \right) - \gamma _{\mathrm{L}}\left( {\bar \theta } \right)$, that counteracts the effect of the total polymer concentration gradient $\nabla c_{\mathrm{P}}$. As the liposomes are swept towards the rear end, the opposing Marangoni force increases (as both *σ* and Δ*γ*(*σ*) increase) and the motion of the droplet is retarded. This behaviour is analogous to the well-known surface immobilisation effect for a rising bubble in a surfactant solution^49–52^. However, once the maximum liposome surface concentration *σ* is achieved, the liposomes are expelled from the droplet interface, due to repulsive electrostatic or steric interactions^40^ and the intensity of the opposing Marangoni force approaches its peak. Finally, the droplet sets into motion while liposomes are ejected from the rear end. As liposomes are removed from the droplet interface, the opposing Marangoni force decreases and the droplet velocity increases towards the peak value *U*_max_, given by Eq. (1) that holds for a droplet with a clean surface, namely without liposomes at the interface (i.e. *θ* = π and *ε* = 1).

The proposed physical description of the droplet motion is consistent with the experimental observations. As shown in Fig. 4b, the droplets do not set into motion as long as the liposomes remain attached to the droplet interface and are accumulated towards the droplet rear end (*ε* > 0) due to the opposing effects of the interfacial tension gradient $\frac{{\partial \,\gamma }}{{\partial \,c_{\mathrm{P}}}}$ and the interfacial tension contrast, Δ*γ*(*σ*). As the liposomes are ejected from the droplet interface, the droplets move at an increasing speed until this reaches a peak value *U*_max_. Figure 4c shows that *U*_max_ increases linearly with the droplet diameter, as predicted by Eq. (1). Furthermore, Eq. (1) predicts, under the examined experimental conditions, a maximum speed of $U_{{\mathrm{max}}} \simeq 6$ µm/s for a 20 µm droplet, which agrees well with the experimental observations (Fig. 4c). Since the opposing Marangoni force is only determined by *ε* (both *σ* and Δ*γ*(*σ*) are solely determined by *ε*), then the droplet velocity only changes with *ε*. We defined the normalised velocity (*U*_R_) as the ratio of droplet velocity at each *ε* to its maximum velocity, and Fig. 4d shows that the *U*_R_–*ε* curve is roughly overlapped for different droplets, fitting well with the theory.

By using scaling arguments, it can be shown that the linear relationship between droplet speed and diameter holds for any droplet velocity, and not just for the peak velocity. Indeed, the force *F*_M_ induced by Marangoni stresses, driven by $\nabla c_{\mathrm{P}}$ and *σ*, is proportional to the droplet surface area, *F*_M_ ∝ *D*^2^, whereas the viscous drag *F*_D_ is proportional to *UD*. From the force balance *F*_M_ = *F*_D_, it follows *U* ∝ *D*. Additionally, for droplets within the same region of the sample, one can expect that they are exposed to the same polymer concentration gradients $\nabla c_{\mathrm{P}}$. If then, droplets have similar values of *ε*, the ratio *U*/*D* should also be similar, and thus the bigger droplets should exhibit a larger velocity. This behaviour is confirmed by Fig. 4e and Supplementary Movie 6 which show the chasing process of a big droplet (droplet A) to a small droplet (droplet B). As shown in the figure, the distance between these two droplets gradually decreases with time during the first 6 s. When droplet A collides into droplet B, they merge into a new larger droplet (droplet C) having two liposome caps located at opposite sides of the droplet interface (9–12 s Fig. 4e). This two-cap liposome distribution gradually turns into a single-cap distribution, driving the transformation of droplet C into a Janus droplet (12–15 s in Fig. 4e). Droplet C is almost stationary during the transformation process, and it starts accelerating as droplet C becomes a Janus droplet (18–24 s in Fig. 4e).

**Fig. 5:**
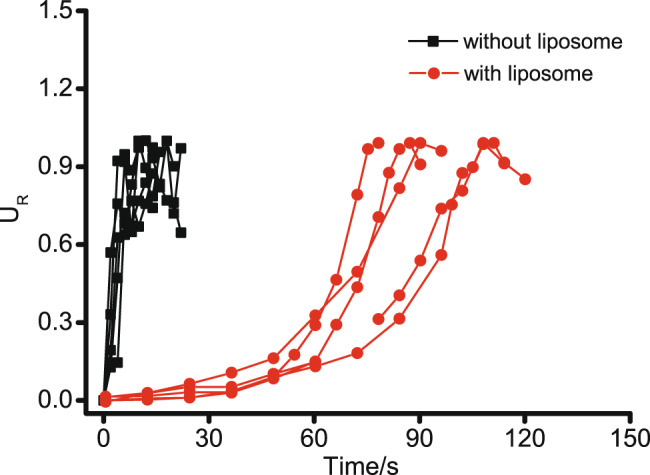
Effect of liposome coating on droplet motion. Emulsion droplets were prepared with/without liposomes, and water was added to induce droplet motion. Five droplets with or without liposome were chosen from different trials for analysis. The ratio of droplet velocity at different time points to its maximum velocity was taken as relative velocity (*U*_R_).

To show the dampening effect of the liposome coating on droplet motion, we also undertook motion experiments without liposomes, preparing PEG/DEX emulsion droplets as previous (but without liposome stabilisers, Supplementary Movie 7). Five droplets were selected to compare with the five droplets in trial 1, and the maximum velocity for these droplets is shown in Supplementary Fig. 8. For each droplet, the ratio of instantaneous velocity at each time point to the maximum velocity was taken as normalised velocity (*U*_R_). As shown in Fig. 5, without a liposome coating the droplet velocity shows a sharp increase, indicating a large acceleration. In comparison, for droplets with a liposome coating, the velocity grows gradually and the onset of this motion is delayed due to the process of Janus formation. These combined effects lead to dramatic differences in the time needed for each droplet species to reach maximum velocity (*t*_max_): when droplets move with liposome coating, *t*_max_ is 94.8 ± 14.2 s (*n* = 5), which is nearly seven times higher than that for droplets without a liposome coating (13.6 ± 4.1 s, *n* = 5). Such results show the dampening effect of liposome coating of emulsion droplets, which can simultaneously stabilise the droplets, as well as help insulate droplet species to the effects of chemical gradients in their local environment.

## Discussion

In summary, we have prepared water-in-water emulsion droplets by mixing liposomes with PEG/DEX ATPS. To generate stable emulsions, PEGylated liposomes are needed to act as droplet stabilisers to prevent coalescence, and the optimal content for PEG lipids in liposomes was found to be 1.5 mol%. In our PEG/DEX emulsion system, emulsion droplets are composed of DEX-rich phase, and intact liposomes stay at the droplet interface to form a liposome coating. With an increase in liposome concentration, there are more liposomes at the droplet interface, increasing droplet stability. In addition, electrostatic repulsion between liposomes increases the surface area that can be covered at a given liposome concentration, which is beneficial to droplet stability. These droplets undergo structural transformation and then directional motion in response to a concentration gradient formed by the addition of water. The trajectory of this motion is strongly directional, occurring down the PEG/DEX polymer gradient (and perpendicular to the cross-section of the liposome coating). During movement, droplet velocity gradually increases to a maximum, which fits well with the theoretical analysis.

Our platform combines the stabilising effect of the interfacial liposome coating, with Marangoni-driven motility and chemotaxis. Importantly, we show the liposomes themselves can act as control elements, and act as dampening modulators to control both the onset of motion, as well as droplet acceleration, making this motile system more controllable. In addition, liposomes are capable of encapsulating content^53–55^ in their lumen, and controllable release can be achieved through the incorporation of photo-responsive content such as diacetylene functional groups^53^ into the liposome composition or membrane proteins such as *α*-hemolysin^54^ or mechanosensitive channels^55^ in lipid bilayers, which can be exploited to expand the application for chemotaxis. The presence of lipid membranes associated with the droplet can also pave the way for the incorporation of membrane-associated biomolecular machinery in future. Finally, given both droplet lumen and outer aqueous environment are cytoplasm-like high viscous environment, our system holds great potential in researching motion-related cellular process. For example, signalling biomolecules can be encapsulated within a droplet lumen and delivered to natural cells located in the outer aqueous environment to trigger specific cellular activities, mimicking intercellular communication.

## Methods

### Materials

PEG 8 kDa was purchased from Fisher Scientific (P1563). Fluorescein isothiocyanate–dextran (FITC-Dextran, FD10S) and Dextran 10 kDa (D9260) were purchased from Sigma Aldrich. 1,2-dioleoyl-sn-glycero-3-phospho-(1′-rac-glycerol) (sodium salt) (DOPG, 840475), 1,2-dioleoyl-sn-glycero-3-phosphoethanolamine-N-[methoxy(polyethylene glycol)-2000] (ammonium salt) (DOPE-PEG2k, 880130), and 1,2-dioleoyl-sn-glycero-3-phosphoethanolamine-N-(lissamine rhodamine B sulfonyl) (ammonium salt) (DOPE-Rh, 810150) were purchased from Avanti Polar Lipids, and all these lipids are dissolved in chloroform. Water was purified via ultrafilter (arium® CellPlus, Sartorius Lab Instruments).

### Preparation of aqueous two-phase system (ATPS)

10 w% PEG, 16 w% Dextran and 74 w% water were mixed in a 50 mL Falcon centrifuge tube and then vortexed at 2500 rpm (Agitateurs Vortex, 444–2790, VWR) until fully dissolved. The mixture was then centrifuged at 3070 × g for 15 min (EBA 21 centrifuge, Hettich) to accelerate the phase separation process. Two clear phases should be seen after centrifugation, and the upper phase is PEG-rich phase while the lower is DEX-rich phase.

For ATPS composition, there are six contents to determine: the PEG content, the DEX content and the water content for PEG-rich phase and DEX-rich phase. Among them, four parameters are free. To determine the four contents, we mixed 2 g PEG, 3.2 g DEX (with 5 wt.% FITC-DEX), and 14.8 g water to prepare PEG/DEX ATPS. After centrifugation, we separated the PEG-rich phase and DEX-rich phase and measured the weight of each phase. Then the FITC fluorescence was measured via a spectrophotometer (Cary Eclipse Fluorescence Spectrophotometer, Agilent Technologies, Inc.) to determine the partition coefficient for DEX in ATPS, which can be used to calculate the DEX content in each phase. For water content, we added 0.5 g DEX-rich phase in each vial and put three vials in a desiccator for evaporation for 2 weeks. By measuring the weight of vial after desiccation, we can calculate the water content for DEX-rich. Then the water content for PEG-rich can be calculated based on the total weight of water in ATPS (14.8 g), the weight of DEX-rich and the water content for DEX-rich.

### Preparation of liposomes

97.5 mol% DOPG, 1.5 mol% DOPE-PEG2k and 1.0 mol% DOPE-Rh (or DOPE-NBD) were mixed in a glass vial (50 × 12 mm, 1481–1582, Fisher Scientific) to form a mixture containing 2.5 mg of lipids. For unPEGylated liposomes, 99 mol% DOPG and 1 mol% DOPE-Rh were used. Chloroform in the mixture was evaporated using a nitrogen stream to form a thin lipid film within the vial, then the vial was put in a vacuum desiccator for at least 2 h to further remove residual chloroform. The anhydrous lipid film was hydrated with 1 mL PEG-rich phase to produce a solution with a lipid concentration of 2.5 mg/mL. This solution was firstly processed with five free-thaw cycles and then extruded with 11 passes through a 0.1 µm filter (Whatman filters, Avanti Mini-Extruder) to produce liposomes. The liposomes were characterised via transmission electron microscopy (JEM-2100F (JEOL, Ltd) fitted with an Orius SC1000 CCD Camera (Gatan, Inc.) and dynamic light scattering (Zetasizer Ultra, Malvern Panalytical), respectively (Supplementary Fig. 9).

### Emulsion stability

A 242.5 μL liposome solution, 727.5 μL PEG-rich phase and 30 μL DEX-rich phase were mixed in a cuvette. For control assay with no liposomes, 970 μL PEG-rich phase and 30 μL DEX-rich were used. The above mixture was mixed using a pipette to achieve thorough mixing, and then the absorbance change was monitored using a UV-visible spectrophotometer (Biochrom Libra S32PC UV/Vis Spectrophotometer, Biochrom Libra Instruments) at 650 nm for 8 h.

### Droplet imaging and analysis

To create a mixture with various liposome concentrations, a 4–16 μL liposome solution and 178–190 μL PEG-rich phase were added into a 0.5 mL centrifuge tube respectively, while the volume of DEX-rich phase was fixed at 6 μL (total mixture volume was 200 μL). The mixture was vortexed at 2500 rpm for 30 s to produce a uniform emulsion. After that, this emulsion was transferred into a chamber (PMMA, 10 mm diameter × 2 mm depth, fabricated via laser cutting platform, ADVL-01039, Universal Laser Systems) for imaging. Images were acquired with an Olympus microscope (IX81, IX2-ILL100, Olympus) or a Nikon microscope (Eclipse TE2000-E, Nikon) in 8-bit type at 1024 × 1024 resolution and saved as.tiff files. The magnification scales varied from 2x to 20x. Rhodamine and NBD filter sets were used, respectively, to measure corresponding fluorescence level. The images were then processed using ImageJ and colour was applied to facilitate data analysis. Merged images were generated via merge channel in ImageJ, and the brightness and contrast were subsequently adjusted for clarity. To analyse droplet stability, we captured a picture at each time point to measure the droplet size (Supplementary Fig. 3). At each time point, 50 droplets were chosen for measurement to obtain the average. All images used were obtained from the fluorescence channel.

### Droplet velocity and liposome coating intensity

Hundred microlitres of 0.10 mg/mL emulsion was transferred into a chamber and left for 1 h to equilibrate. Then, 50 μL of pure water was added into this chamber to induce droplet motion. Olympus microscope (IX81, IX2-ILL100, Olympus) or Nikon microscope (Eclipse TE2000-E, Nikon) was used to monitor the whole process. For recording, image stacks were acquired in 8-bit type at 1024 × 1024 resolution and saved as .tiff files. Droplets trajectory and velocity were measured using ImageJ (Fiji ImageJ>Plugins>Tracking>Manual Tracking).

To measure the fluorescence intensity of liposome coatings, a concentric circle equivalent to the droplet diameter was drawn to encircle the droplet, and that circle then was added into ROI manager for measurement (Fiji ImageJ>Analyze>Tools>ROI manager>Measure). The measurement result includes circle area, mean intensity, minimum intensity and maximum intensity. For each droplet, the difference between mean intensity and minimum intensity can be seen as the intensity of liposome coating. The height of the dark droplet part (droplet surface without liposome coating) was measured manually by drawing auxiliary lines (Fig. 3d).

## Supplementary information

Supplementary Information

Description of Additional Supplementary Files

Supplementary Movie 1

Supplementary Movie 2

Supplementary Movie 3

Supplementary Movie 4

Supplementary Movie 5

Supplementary Movie 6

Supplementary Movie 7

## Data Availability

The authors declare that the data supporting the findings of this study are available within the paper and its supplementary information files. Data is available from the corresponding author upon reasonable request.
