# Supplementary material for: Engineering motile aqueous phase-separated droplets via liposome stabilisation
Source: Nat Commun. 2021 Mar 15;12:1673. doi: 10.1038/s41467-021-21832-x (PMC7960712; doi:10.1038/s41467-021-21832-x)
Supplement: Supplementary file 1 — Supplementary Information [file 41467_2021_21832_MOESM1_ESM.pdf]

Supplementary Information

Engineering motile aqueous phase-separated droplets via liposome stabilisation

Shaobin Zhang[1], Claudia Contini[2,3], James W. Hindley[1,3,4], Guido Bolognesi[5], Yuval Elani[2,3,4], Oscar Ces[1,3,4]*

1. Department of Chemistry, Imperial College London, Molecular Sciences Research Hub, Shepherd's Bush, London, W12 0BZ, UK
2. Department of Chemical Engineering, Imperial College London, South Kensington, London, SW7 2AZ, UK.
3. fabriCELL, Imperial College London, Molecular Sciences Research Hub, Shepherd's Bush, London, W12 0BZ, UK
4. Institute of Chemical Biology, Imperial College London, Molecular Sciences Research Hub, Shepherd's Bush, London, W12 0BZ, UK
5. Department of Chemical Engineering, Loughborough University, Loughborough, LE11 3TU, UK

* Correspondence: o.ces@imperial.ac.uk

1 ATPS composition

Supplementary Table 1 Composition of two phases in ATPS

Phase	PEG content (%w)	DEX content (%w)	water content (%w)
**PEG-rich phase**	17.1±3.8	4.8±0.3	78.1±3.5
**DEX-rich phase**	0.8±0.6	30.5±0.5	68.7±0.1

2 Emulsion stability

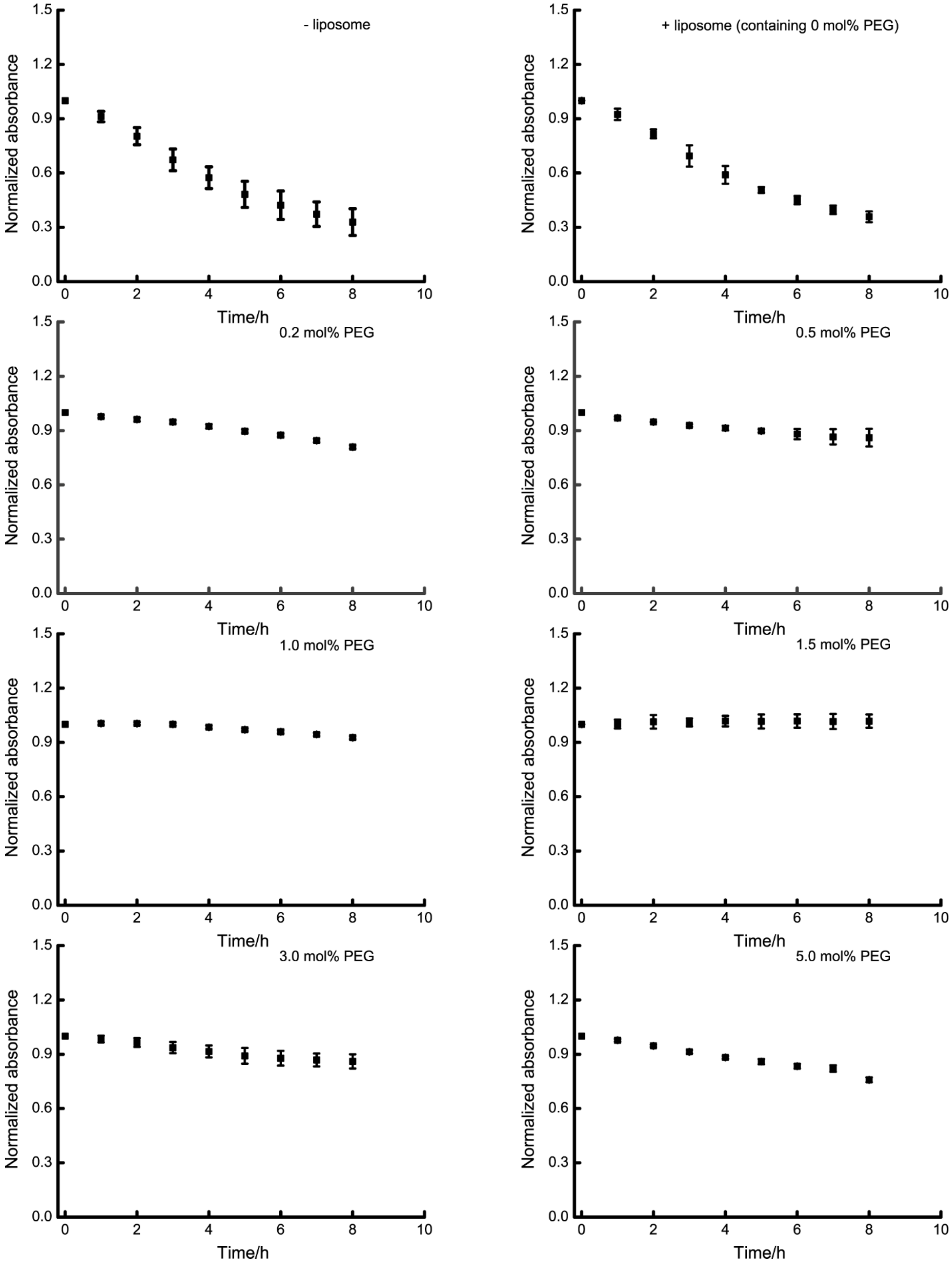

Supplementary Fig.1 Emulsion absorbance change during 8 h. If emulsions stay stable, it should remain turbid during observation, i.e. the absorbance remain unchanged. Otherwise a decrease in absorbance can be observed for unstable emulsions. Final absorbance at 8 h was extracted for quantitative analysis, which is shown in Fig.2(a). Error bars represent 1 s.d. (n = 3)

3 Size exclusion chromatography

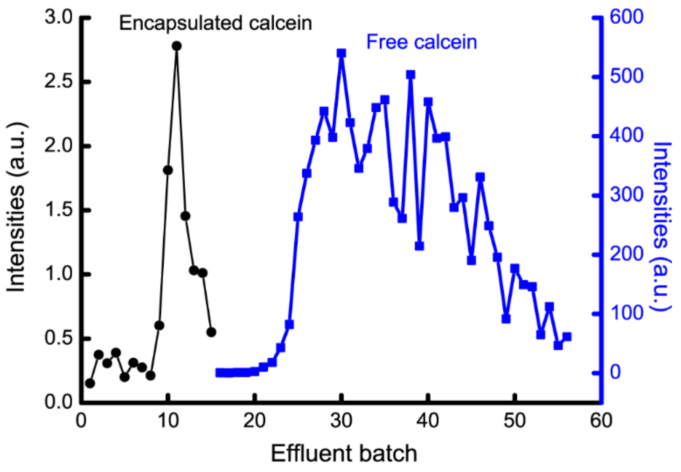

Supplementary Fig.2 Intensity for different effluent batches in size chromatography experiment. Two peaks can be observed, and the first peak is corresponding to encapsulated calcein, while the second is for free calcein.

4 Droplet stability

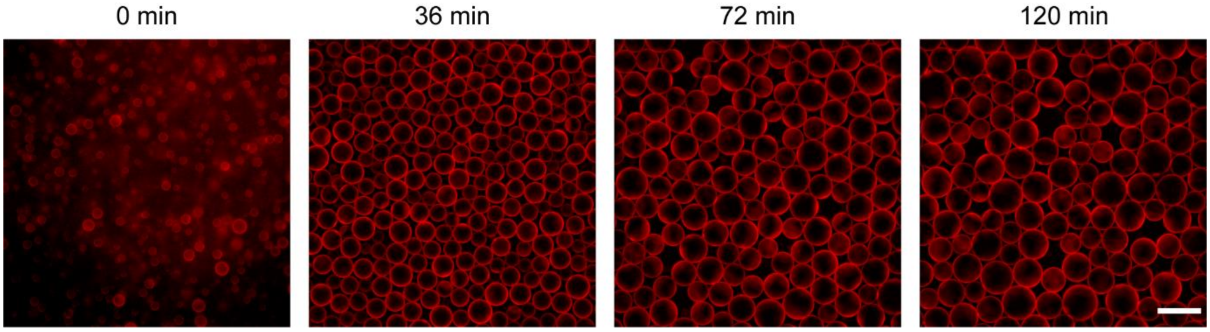

Supplementary Fig. 3 Droplet size at different time points. Liposome concentration is 0.10 mg/mL. From 0 min to 120 min, droplet size first gradually increases due to droplet merge, then limited change in droplet size occurs after liposome coverage is enough to stabilize emulsion droplets. Scale bar is 50 μm.

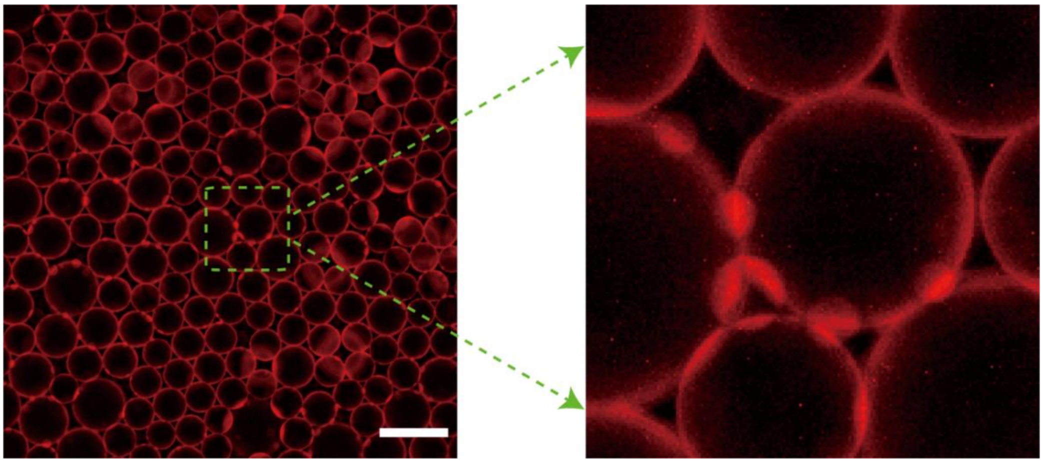

Supplementary Fig.4 Effect of adhesion/superimpose on droplet morphology. Due to the superimpose/adhesion of small droplets to big droplets, some bright dots can be observed at droplet surface, which makes the liposome coating for big droplets is seemingly heterogenous. Scale bar is 50 μm.

The equation used to calculate the number of lipid molecules in a unilamellar liposome can be written as follows:

$$N = \frac{4\pi\left(\frac{d}{2}\right)^2 + 4\pi\left(\frac{d}{2} - h\right)^2}{a} \tag{1}$$

Where d is the liposome diameter, h is the bilayer thickness (about 5 nm), a is the area of lipid head group.

According to Supplementary equation (1), we can calculate that the lipid molecules contained in a 150.5 nm liposome is approximate 2.23 times of that for a 102.3 nm liposome. Thus, to prepare the same number of liposomes, the lipid concentration needed for 150.5 nm liposomes is 2.23 times of that for 102.3 nm liposomes, i.e., 0.10 mg/mL (150.5 nm) versus 0.045 mg/mL (102.3 nm).

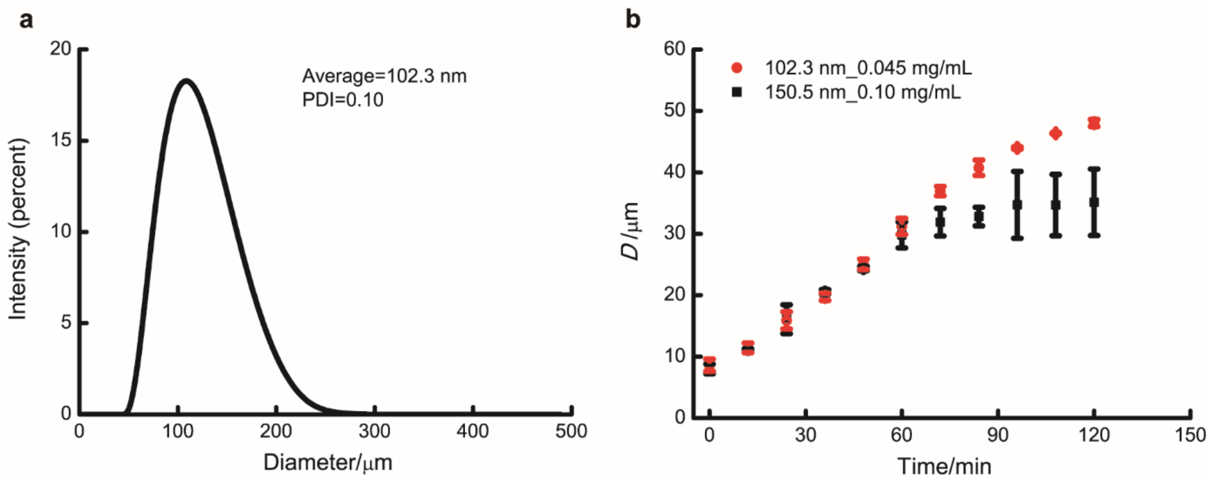

Supplementary Fig. 5 Effect of liposome size on droplet stability. (a) Size distribution for liposomes prepared via 50 nm filter. PDI is Polydispersity Index. **(b)** Effect of liposome size on droplet stability. *D* is droplet diameter. At the same liposome quantity (0.10 mg/mL for 150.5 nm versus 0.045 mg/mL for 102.3 nm), larger liposomes correspond to better droplet stability than smaller liposomes as larger liposomes can cover a larger surface area. Error bars represent 1 s.d. (n = 3)

5 Liposome surface concentration

Emulsion droplets are produced via vortex, and the initial average droplet size is only dependent on vortex strength (shear force), i.e., initial average droplet size is approximately constant once the vortex strength is fixed. Since the volume of DEX-rich phase is fixed at 6 μL, thus the initial number of emulsion droplets is approximately constant. At the same liposome concentration, the initial number of liposomes in the liposome coating (i.e., liposome surface concentration) then should be approximately the same. We can consider these droplets as unit droplets that create new droplets via droplet merging. The volume of a new droplet should be integer multiples to that of unit droplet.

For a unit droplet,

$$N_{\mathrm{unit}} = \sigma_{\mathrm{unit}} A_{\mathrm{unit}} \tag{2}$$

Where N_{unit} is the initial number of liposomes in a liposome coating, A_{unit} is the initial droplet surface area, and σ_{unit} is the initial surface concentration for liposomes on droplet's surface.

Consider a droplet generated via the merge of n unit droplets,

$$N_1 = \sigma_1 A_1 \tag{3}$$

Where N_1 is the number of liposomes in a liposome coating, A_1 is droplet surface area, and σ_1 is liposome surface concentration.

The volume for this new droplet (V_1) can be calculated as below,

$$V_1 = nV_{\mathrm{unit}} = \frac{\pi {D_1}^3}{6} = \frac{n\pi {D_{\mathrm{unit}}}^3}{6}$$

$$(4)$$

Where D_{unit} and D_1 is the diameter for unit droplet and the new droplet respectively.

Thus, the surface area for this new droplet can be calculated as below,

$$A_1 = \pi\left(\frac{6V_1}{\pi}\right)^{\frac{2}{3}} = n^{\frac{2}{3}}\pi D_{\mathrm{unit}}{}^2 \tag{5}$$

Then σ_1 can be calculated as below,

$$\sigma_1 = \frac{N_1}{A_1} = \frac{nN_{\mathrm{unit}}}{A_1} = \frac{n\sigma_{\mathrm{unit}}A_{\mathrm{unit}}}{A_1} = n^{\frac{1}{3}}\sigma_{\mathrm{unit}} \tag{6}$$

Combine supplementary equation (4) and (6),

$$\sigma_1 = n^{\frac{1}{3}}\sigma_{\mathrm{unit}} = \frac{D_1\sigma_{\mathrm{unit}}}{D_{\mathrm{unit}}} = kD_1 \tag{7}$$

Where $k = \frac{\sigma_{\mathrm{unit}}}{D_{\mathrm{unit}}}$ is a constant for droplets. Supplementary equation (7) means that liposome surface concentration is proportional to droplet diameter.

6 Fluorescence intensity change

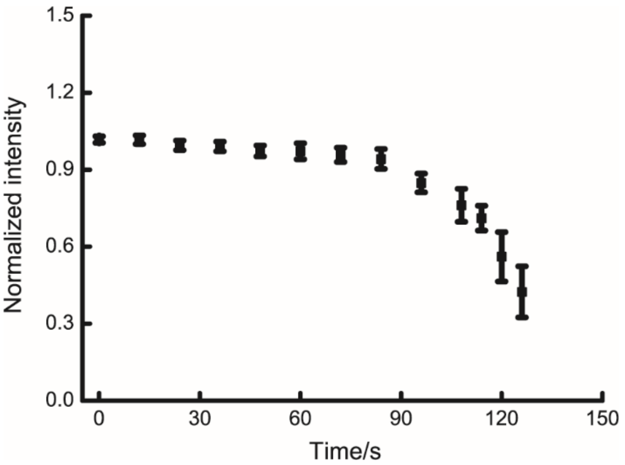

Supplementary Fig.6 Intensity change during Janus transformation and droplet motion. Before droplet motion, the normalized intensity of droplets keeps nearly constant (the plateau stage), which means no loss of liposome. When droplet starts moving, the intensity will gradually decrease due to the loss of liposomes caused by surface flow. Error bars represent 1 s.d. (n = 4)

7 Effect of PEG-rich addition and DEX-rich addition

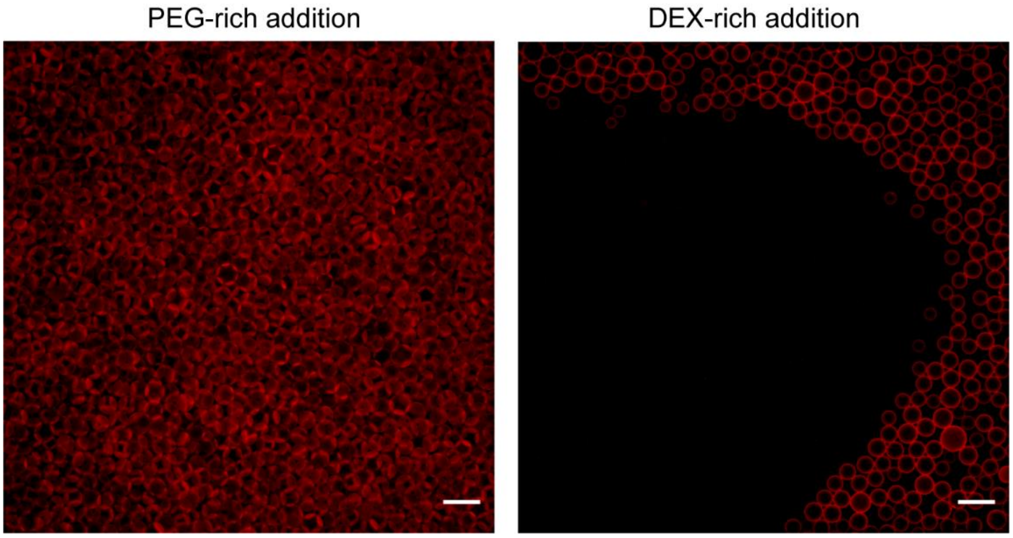

Supplementary Fig.7 Effect of PEG-rich addition and DEX-rich addition. PEG-rich addition simply causes limited droplet merging within the local vicinity, while adding more of the DEX-rich phase eliminates a great number of emulsion droplets within the chamber. Scale bar is 50 μm.

8 Theoretical model for droplet motion

For a qualitative analysis of droplet motion, we can model the droplet surface as a sharp (not diffuse) interface with an IFT $\gamma(c_\mathrm{P},\sigma)$ that depends on the total polymer concentration c_P and the surface concentration σ. Cartesian $(\mathbf{e}_x,\mathbf{e}_y,\mathbf{e}_z)$ and spherical $(\mathbf{e}_r,\mathbf{e}_\theta,\mathbf{e}_\phi)$ coordinate systems are defined with the origin at the droplet centre and z axis parallel to the total polymer concentration gradient, $\nabla c_\mathrm{P} = \frac{\mathrm{d}c_\mathrm{P}}{\mathrm{d}z}\,\mathbf{e}_z$. The IFT gradient at the droplet interface (i.e., at $r = R$ with R the droplet radius) can be written as

$$\nabla\gamma = \left(-\sin\theta\frac{\mathrm{d}c_\mathrm{P}}{\mathrm{d}z}\frac{\partial\gamma}{\partial c_\mathrm{P}} + \frac{1}{R}\frac{\partial\gamma}{\partial\sigma}\frac{\partial\sigma}{\partial\theta}\right)\mathbf{e}_\theta \tag{8}$$

Such a gradient induces the droplet motion by Marangoni effect at speed $\mathbf{U} = U\,\mathbf{e}_z$. To determine the expression of U, the Stokes equations must be solved[1,2]

$$\nabla\cdot\mathbf{u}^{(\alpha)} = 0 \tag{9}$$

$$\mu_\alpha\,\nabla^2\mathbf{u}^{(\alpha)} = \nabla\,p^{(\alpha)} \tag{10}$$

with $\mathbf{u}^{(\alpha)}$, $p^{(\alpha)}$, μ_α represent the velocity, pressure and viscosity, respectively, of the inner ($\alpha = \mathrm{i}$) and outer ($\alpha = \mathrm{o}$) liquid phase. Due to the symmetry of the problem[3], the velocity fields can be expressed in term of stream functions $\psi^{(\alpha)}$, such that

$$u_\theta^{(\alpha)} = -\frac{1}{r\sin^2\theta}\frac{\partial\psi^{(\alpha)}}{\partial\theta}\ , \quad u_r^{(\alpha)} = \frac{1}{r\sin\theta}\frac{\partial\psi^{(\alpha)}}{\partial r} \tag{11}$$

with $\mathbf{u}^{(\alpha)} = u_r^{(\alpha)}\mathbf{e}_r + u_\theta^{(\alpha)}\mathbf{e}_\theta$. For the sake of simplicity, we neglect the effects of liposomes on the droplet interface, so that the IFT gradient can be re-written as

$$\nabla\gamma = -\sin\theta \frac{\mathrm{d}c_P}{\mathrm{d}z}\frac{\mathrm{d}\gamma}{\mathrm{d}c_P}\mathbf{e}_\theta \tag{12}$$

If we also assume that both $\frac{\mathrm{d}c_\mathrm{P}}{\mathrm{d}z}$ and $\frac{\partial\gamma}{\partial c_\mathrm{P}}$ are constant, the stream functions $\psi^{(\alpha)}$ can be written as

$$\psi^{(\alpha)} = \sin^2\theta\left(\frac{A^{(\alpha)}\,r^4}{10} - \frac{B^{(\alpha)}\,r}{2} + C^{(\alpha)}r^2 + \frac{D^{(\alpha)}}{r}\right) \tag{13}$$

The eight arbitrary constant $A^{(\alpha)}, B^{(\alpha)}, C^{(\alpha)}, D^{(\alpha)}$ are determined by imposing the following boundary conditions

$$u_r^{(\mathrm{o})} \rightarrow -U\cos\theta,\quad u_\vartheta^{(\mathrm{o})} \rightarrow U\sin\theta \qquad \text{for} \qquad r \rightarrow \infty,$$

$$u_r^{(\mathrm{o})} = u_r^{(\mathrm{i})} = 0, \qquad u_\theta^{(\mathrm{o})} = u_\theta^{(\mathrm{i})} \quad \text{for} \qquad r = R,$$

$$p^{(\mathrm{i})} = p^{(\mathrm{o})} + \frac{2\gamma}{R},\quad \tau_{r\theta}^{(\mathrm{i})} - \tau_{r\theta}^{(\mathrm{o})} = \nabla\gamma\cdot\mathbf{e}_\theta \quad \text{for} \qquad r = R,$$

$$u_\theta^{(\mathrm{i})}\ ,\ u_r^{(\mathrm{i})} \quad \text{finite for} \qquad r \rightarrow 0 \tag{14}$$

with the tangential stresses at the droplet interface given by

$$\tau_{r\theta}^{(\alpha)} = \mu_\alpha\left(\left.\frac{\partial u_\theta^{(\alpha)}}{\partial r}\right|_{r=R} - \frac{u_\theta^{(\alpha)}(r=R)}{R}\right)$$

(15)

From Supplementary equation (14), one can obtain

$$A^{(\mathrm{o})} = 0, \;\; B^{(\mathrm{o})} = \frac{R}{(\mu_\mathrm{o} + \mu_\mathrm{i})}\left[\frac{U}{2}(3\mu_\mathrm{i} + 2\mu_\mathrm{o}) + \frac{R}{3}\;\frac{\mathrm{d}c_\mathrm{P}}{\mathrm{d}z}\;\frac{\mathrm{d}\gamma}{\mathrm{d}c_\mathrm{P}}\right],$$

$$C^{(\mathrm{o})} = 0\,, \;\; D^{(\mathrm{o})} = \frac{R^3}{2(\mu_\mathrm{i} + \mu_\mathrm{o})}\left(\frac{U\mu_\mathrm{i}}{2} + \frac{R}{3}\frac{\mathrm{d}c_\mathrm{P}}{\mathrm{d}z}\;\frac{\mathrm{d}\gamma}{\mathrm{d}c_\mathrm{P}}\right)$$

$$A^{(\mathrm{i})} = \frac{5}{3\,R\,(\mu_\mathrm{i} + \mu_\mathrm{o})}\left(\frac{3U\mu_\mathrm{o}}{2\,R} - \frac{\mathrm{d}c_\mathrm{P}}{\mathrm{d}z}\;\frac{\mathrm{d}\gamma}{\mathrm{d}c_\mathrm{P}}\right), \;\; B^{(\mathrm{i})} = 0,$$

$$C^{(\mathrm{i})} = -\frac{R}{6\,(\mu_\mathrm{i} + \mu_\mathrm{o})}\left(\frac{3U\mu_\mathrm{o}}{2\,R} - \frac{\mathrm{d}c_\mathrm{P}}{\mathrm{d}z}\;\frac{\mathrm{d}\gamma}{\mathrm{d}c_\mathrm{P}}\right), \;\; D^{(\mathrm{i})} = 0$$

(16)

Due to the symmetry of the problem[3], the total force exerted by the fluid on the droplet can be expressed as

$$\mathbf{F} = F\,\mathbf{e}_z = -4\,\pi\,\mu_\mathrm{o}\,B^{(\mathrm{o})}\,\mathbf{e}_z$$

(17)

By casting the expression of $B^{(\mathrm{o})}$ in Supplementary equation (17), we get the expression for the force F

$$F = \frac{-4\,\pi\,\mu_o\,R}{\mu_o + \mu_i}\;\left[\frac{U}{2}(3\mu_i + 2\mu_o) + \frac{R}{3}\;\frac{\mathrm{d}c_P}{\mathrm{d}z}\;\frac{\mathrm{d}\gamma}{\mathrm{d}c_P}\right]$$

(18)

In absence of the IFT gradient, Supplementary equation (18) reduces to the known expression[3] for the drag force on a droplet moving at constant speed U, and, by taking $\mu^{(\mathrm{i})} \to \infty$, it further reduces to the know expression for the drag force on a solid particle, namely $F = 6\,\pi\,\mu^{(\mathrm{o})}\,R\,U$.

By imposing the force balance on the droplet ($F = 0$), the following expression of U can be obtained

$$U = \frac{-D}{3\,(2\mu_o + 3\mu_i)} \frac{\mathrm{d}c_P}{\mathrm{d}z} \frac{\mathrm{d}\gamma}{\mathrm{d}c_P} \tag{19}$$

with $D = 2\,R$, the droplet diameter. It is worth noting that this expression matches that one obtained by Levan and Newman[1] when $\frac{\mathrm{d}\gamma}{\mathrm{d}z}$ is constant and the gravity effect is neglected.

The proposed model has a number of limitations. First of all, the effect of liposomes adsorbed to the droplet interface is not accounted for. Furthermore, the dynamics of PEG and DEX polymers within the outer and inner phases is neglected and, for sake of simplicity, a constant total polymer concentration gradient $\frac{\mathrm{d}c_P}{\mathrm{d}z}$ is considered. Time-dependent effects on droplet dynamics are also neglected and only droplet motion at steady-state is considered. Finally, the water flow through the droplet interface was neglected and the boundary conditions of flow impenetrability was used instead. Despite these limitations, the proposed model still captures the basic physical mechanisms governing the droplet motion and the predicted droplet behaviour is consistent with our experimental observations.

Supplementary equation (19) can be used to estimate the order of magnitude of the maximum droplet speed under our experimental conditions. The term $\frac{\mathrm{d}\gamma}{\mathrm{d}c_P}$ is estimated by using spinning droplet tensiometer measurements, reported in Liu et al[4], for PEG and DEX solutions under varying total polymer concentration. According to Liu et al[4], the relation between γ and c_P can be written as

$$\gamma = \bar{\gamma} \left(\frac{c_P}{c_{P,\mathrm{cr}}} - 1 \right)^m$$

(20)

from which follows

$$\frac{\mathrm{d}\gamma}{\mathrm{d}c_P} = \frac{m\,\bar{\gamma}}{c_{P,\mathrm{cr}}}\left(\frac{c_P}{c_{P,\mathrm{cr}}} - 1\right)^{m-1}$$

(21)

with $c_{\mathrm{P,cr}} = 0.05$ g/mL, $m = 1.67$ and $\bar{\gamma} = 146$ μN/m. For our experiment conditions ($c_P \simeq$ 0.3 g/mL) , we can estimate $\frac{\mathrm{d}\gamma}{\mathrm{d}c_P} \simeq 14 \times 10^{-6}$ N m^2 kg^{-1} and $\frac{\mathrm{d}c_P}{\mathrm{d}z} \simeq \frac{0.3\frac{\mathrm{g}}{\mathrm{mL}}}{1\ \mathrm{cm}} \simeq 0.3 \times 10^6$ kg m^{-4}. If we assume a viscosity of 100 cSt for both PEG and DEX phases, Supplementary equation (19) predicts a velocity of ca. 6 μm/s for a droplet of diameter $D = 20$ μm.

9 The maximum velocity for droplets in Fig.5

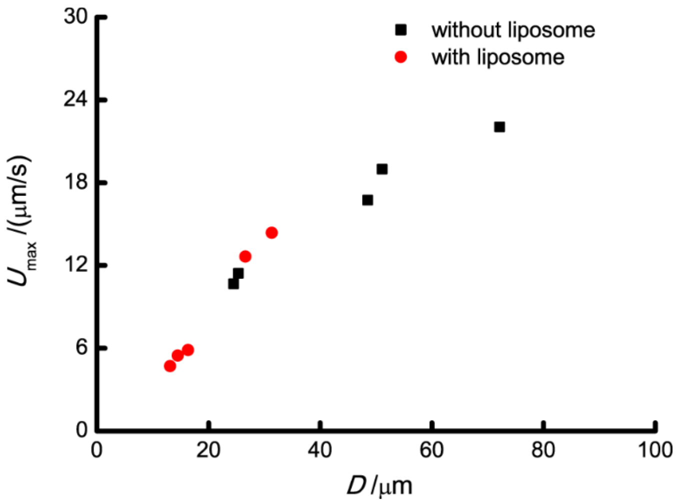

Supplementary Fig.8 The maximum velocity for droplets in Fig.5. Emulsion droplets were prepared with/without liposomes, and water was added to induce droplet motion. 5 droplets with or without liposome were chosen from different trials for analysis. *U*max and *D* is the maximum velocity and droplet diameter respectively.

10 Characterization of liposomes

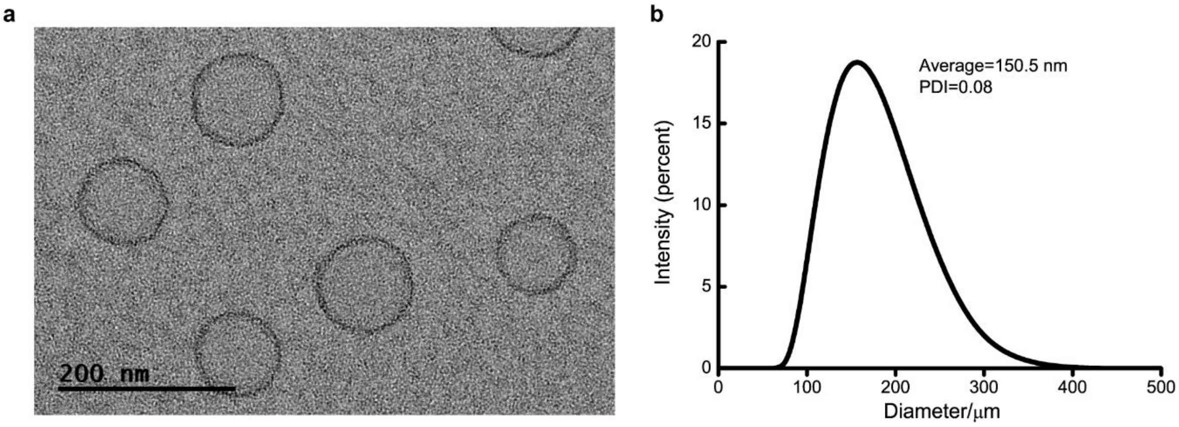

Supplementary Fig.9 liposome structure and size distribution. (a) TEM picture for prepared liposomes. The prepared liposomes are unilamellar. **(b)** Size distribution for prepared liposomes. The average diameter of liposomes is 150.5 nm, which is consistent with filter size used for extrusion. PDI is Polydispersity Index.

Discussion

In our work, the droplet motion is caused by the Marangoni effect derived from the IFT gradient. The IFT gradient is formed by introducing a polymer gradient. Thus, the direction of the IFT gradient is parallel to the direction of polymer gradient. In the model theory part, we show that the motion direction is determined by the direction of the IFT gradient. Droplets in the same batch experience the same polymer gradient, thus the movement direction for these droplets is the same, i.e., the observed directional motion is nearly parallel.

There are several pieces of evidence to show that the motion is not caused by a convective flow. (1) The maximum velocity of droplets increases with droplet diameter, a result which cannot be justified by a droplet motion induced by a convective flow. (2) For example, in Supplementary Movie 1, it can be observed that big droplets have a larger acceleration (and velocity) and will outstrip small droplets in front of it, which illustrates the droplet motion is not caused by convective flow. Also, the onset of particle motion is always preceded by the motion of the droplet-associated liposomes along the droplet interface towards the rear end of the droplets. Such a motion of liposome has opposite direction to the motion of the droplets

and it cannot be induced by a convective flow. Additionally, the formation of a fluorescent tail caused by liposome desorption at the rear end of the moving droplets suggests that the droplets moves at a higher speed than the surrounding liquid, which is incompatible with a droplet motion driven by a convective flow.

To conclude, although it cannot be ruled out that convective flows could possibly affect the droplet motion, our experimental observations show that the observed droplet motion is not caused by convective flows.

References

1. Leven MD, Newman J. The effect of surfactant on the terminal and interfacial velocities of a bubble or drop. *AIChE J* **22**, 695-701 (1976).
2. Levan MD. Motion of a droplet with a Newtonian interface. *J Colloid Interface Sci* **83**, 11-17 (1981).
3. Happel J, Brenner H. (2012). *Low Reynolds number hydrodynamics: with special applications to particulate media* (Vol. 1). Springer Science & Business Media.
4. Liu Y, Lipowsky R, Dimova R. Concentration dependence of the interfacial tension for aqueous two-phase polymer solutions of dextran and polyethylene glycol. *Langmuir* **28**, 3831-3839 (2012).
