# Supplementary material for: Engineering motile aqueous phase-separated droplets via liposome stabilisation
Source: Nat Commun. 2021 Mar 15;12:1673. doi: 10.1038/s41467-021-21832-x (PMC7960712; doi:10.1038/s41467-021-21832-x)
Supplement: Supplementary file 2 — Description of Additional Supplementary Files [file 41467_2021_21832_MOESM2_ESM.pdf]

Description of Additional Supplementary Files

Title: Supplementary Movie 1

Description: Upward droplet motion

Title: Supplementary Movie 2

Description: Leftward droplet motion

Title: Supplementary Movie 3

Description: Downward droplet motion

Title: Supplementary Movie 4

Description: Addition of PEG-rich phase into the emulsion system.

Title: Supplementary Movie 5

Description: Addition of DEX-rich phase into the emulsion system.

Title: Supplementary Movie 6

Description: Chasing process of two moving droplets.

Title: Supplementary Movie 7

Description: Droplet motion without liposomes
